# Interventions to Reduce Burnout Among University Lecturers: A Systematic Literature Review

**DOI:** 10.3390/bs15050649

**Published:** 2025-05-10

**Authors:** Beibei Cao, Norlizah Che Hassan, Muhd Khaizer Omar

**Affiliations:** Faculty of Educational Studies, Universiti Putra Malaysia, Serdang 43400, Selangor, Malaysia; gs62619@student.upm.edu.my (B.C.); khaizer@upm.edu.my (M.K.O.)

**Keywords:** teachers, burnout, interventions, university lecturers

## Abstract

The teaching profession is widely recognized as highly challenging due to its intense workload, emotional demands, and ongoing stressors. This Systematic Literature Review (SLR) aims to identify and evaluate various interventions that have been implemented to address lecturer burnout over the past five years. A thorough review of 21 studies published between 2020 and 2024 was conducted across five major databases: Web of Science, Scopus, PubMed, ERIC, and APA PsycINFO. Relevant search terms were used to explore the effectiveness of different interventions aimed at reducing lecturer burnout. Articles were extracted, reviewed, collated, and thematically analyzed to synthesize the findings. Seven distinct interventions were identified as effective in reducing burnout. The most commonly studied intervention was social support, followed by training programs. Other interventions demonstrating positive results include supportive work environments, Rational Emotive Behavior Therapy (REBT), and psychological capital. Additionally, interventions that balanced work and life conditions, facilitated teaching transitions, helped lecturers reevaluate major work demands, and encouraged the utilization of character strengths were also found to yield beneficial outcomes. The implementation of targeted, school-based interventions is crucial for reducing burnout and enhancing the overall well-being of university lecturers. Policymakers, administrators, and educational leaders should prioritize the implementation of school-based awareness and intervention programs.

## 1. Introduction

Teaching is commonly recognized as one of the most demanding and stressful professions, with burnout becoming a significant and urgent concern. College lecturers, in particular, face increased pressure and heightened expectations from universities, students, and society. The long working hours, coupled with minimal recognition and support, make educators especially susceptible to burnout. Lecturers are tasked with fulfilling a range of responsibilities, including preparing and adhering to curricula, managing extensive administrative work, and meeting the demands of school administrators. In an international comparison of burnout levels, Chinese lecturers reported the second-highest degree of depression among their counterparts from 35 other countries (García-Arroyo & Segovia, 2019).

It is essential to consider the cultural context of China’s higher education system, which differs significantly from that of Western countries. Chinese universities operate within a Confucian-heritage culture that places strong emphasis on hierarchy, respect for authority, and collective responsibility. These cultural norms may shape how work stress is experienced and how social support is sought or perceived by lecturers. In China alone, approximately 1.6 million educators are affected by burnout, drawing considerable attention from researchers (H. Cheng et al., 2023). Given the scale of this issue, addressing burnout among educators has become a critical area of focus in educational research.

Burnout is described as “a psychological syndrome brought on by extended exposure to ongoing interpersonal stressors at work” (Maslach & Leiter, 2016, p. 103). Typically, burnout manifests in three dimensions: emotional exhaustion, depersonalization, and reduced personal accomplishment (Maslach et al., 2001). Numerous organizational and work-related factors, such as length of teaching, class size, job satisfaction, and subject specialization, as well as sociodemographic factors such as age, gender, marital status, and educational background all have an impact on teacher burnout. High levels of burnout have been linked to negative physical and mental health outcomes (Khamisa et al., 2016; Shao et al., 2020; Agyapong et al., 2022). These health issues can, in turn, negatively impact job performance (Song, 2019; Mohammed et al., 2020), reduce job satisfaction (Boamah et al., 2017; Alqassim et al., 2022), increase turnover rates (Alqassim et al., 2022), and decrease student learning and achievement (Matos et al., 2022). Additionally, burnout can diminish organizational effectiveness (Mohammed et al., 2020). In severe cases, it may even lead to early retirement or, in extreme situations, suicide (Christopher et al., 2020; Queirós et al., 2020; Harvey et al., 2021). Given the significant negative impacts of burnout on lecturers, it is crucial to examine and assess the interventions aimed at addressing these psychological issues among university lecturers.

## 2. Related Literature and Research Perspective

### 2.1. Burnout

Burnout is a psychological response to workplace stress (Maslach & Jackson, 1982; Maslach et al., 2001). It is commonly defined by three key dimensions: emotional exhaustion, depersonalization, and reduced personal accomplishment. Anger, frustration, and sadness are all common symptoms of emotional exhaustion. Depersonalization is typified by an impersonal, detached attitude toward other people, frequently viewing them more as things than as unique people. In the workplace, a lower sense of self-efficacy and a negative self-evaluation are examples of reduced personal accomplishment (Wills, 1991).

In the context of Chinese higher education, university lecturers occupy a distinct and often understudied professional role. Compared to adjunct faculty, lecturers typically enjoy greater employment stability, yet they may lack the academic autonomy and research opportunities afforded to tenured or tenure-track faculty. While full professors often engage in research and policy-level decision-making, lecturers are more likely to be evaluated primarily on their teaching performance and face regular contract renewals, which can result in heightened job insecurity and emotional fatigue.

Furthermore, not all lecturers are required to take on administrative responsibilities, and assumptions regarding such duties should be made with caution. The scope of a lecturer’s responsibilities may vary significantly depending on institutional context—some may focus exclusively on teaching, while others are expected to contribute to departmental service or research. This role ambiguity and variability in expectations can further contribute to stress and the risk of burnout.

Additionally, the global COVID-19 pandemic introduced unprecedented challenges that further intensified burnout among university lecturers. Abrupt transitions to online teaching, the need for rapid technological adaptation, heightened health-related anxiety, and increased student support responsibilities all contributed to elevated stress levels during this period (Castro et al., 2023; Padmanabhanunni & Pretorius, 2023; Ozamiz-Etxebarria et al., 2023). Although the present study does not focus exclusively on the pandemic, several referenced works were conducted during this time and reflect the exceptional pressures of that historical context. In China, these challenges were often compounded by policy uncertainty and limited institutional communication, further underscoring the importance of social support as a critical buffer against burnout.

Similar burnout trends have been observed in other high-stress professional domains, particularly in healthcare. Sipos et al. (2024) discuss how burnout in the healthcare workforce—driven by increased workloads, emotional fatigue, and lack of institutional support—was significantly intensified during the COVID-19 pandemic. These findings highlight a shared vulnerability across professions, where insufficient social and organizational support can exacerbate mental health challenges. Drawing parallels with university lecturers, the study emphasizes that effective burnout mitigation must involve targeted interventions at both individual and institutional levels. These cross-sector insights further reinforce the critical need for comprehensive support systems in higher education.

### 2.2. Interventions to Reduce Burnout

Research has consistently identified several interventions that can help reduce burnout among teachers in educational settings. A significant body of research suggests that social support is an effective strategy for alleviating burnout. For example, social support has been found to significantly moderate the associations between stressors and mental health among middle school teachers (Gan, 2022). Additionally, perceived social support has been shown to partially mediate the connections between work stress and job burnout among elementary and middle school teachers (Zhao & Chen, 2020). Taylor and Frechette (2022) discovered that perceived social support negatively correlates with burnout, helping to alleviate work stress and reduce high burnout levels among faculty members. Furthermore, support from friends, relatives, co-workers, and even pets has proven beneficial for university lecturers in managing work stress and preventing burnout in higher education (Kolomitro et al., 2020). Therefore, social support has emerged as a key strategy that can reduce burnout by alleviating work stress.

A systematic review by von der Embse et al. (2019), which included 24 studies published between 1998 and 2017, Mindfulness-Based Interventions (MBIs), Cognitive-Behavioral Therapy (CBT) and behavioral interventions were the most effective. However, the least effective interventions were those that only provided informational content. Another review by Hagermoser Sanetti et al. (2021), covering 18 studies published from 1987 to 2016, found that meditation and mindfulness-based techniques were the most often assessed stress-reduction strategies. A more recent scoping review (2018–2022) examined interventions aimed at reducing burnout among lecturers and identified several effective approaches, including the following: MBIs, often combined with yoga or in combination with CBT; Rational Emotive Behavior Therapy (REBT); Christian prayer and prayer-reflection; group sandplay; stress reduction training; sports-based physical activity programs; and building social and emotional competence (Agyapong et al., 2023). Furthermore, Hidajat et al. (2023) offer an updated synthesis of Mindfulness-Based Interventions (MBIs), which included 39 studies published between 10 and 14 November 2021, aimed at addressing stress and burnout among lecturers. The evidence from these interventions demonstrates significant potential for reducing stress and burnout, as well as for improving other key psychological outcomes.

### 2.3. Knowledge of Gaps and Aims of Review

While extensive research has highlighted the effectiveness of various interventions—such as social support, MBIs, CBT, and other therapeutic approaches—in reducing burnout among lecturers, several key gaps remain. Notwithstanding these developments, thorough and systematic reviews are required that synthesize the achievements of the preceding five years and pinpoint areas in need of additional study. Furthermore, there is an absence of Systematic Literature Reviews (SLRs) on interventions aimed at reducing lecturers’ burnout. SLRs are essential for assessing the present level of the field on a specific subject, as they provide a thorough summary of the available data and help guide future investigations (Whittemore & Knafl, 2005; Liberati et al., 2009). To address the existing research gap, this review aims to offer a thorough and up-to-date summary of empirical studies on interventions for reducing burnout among university lecturers. It focuses on research published in peer-reviewed, English-language journals worldwide between 2020 and 2024.

This review aims to identify and summarize the various interventions designed specifically for university lecturers to reduce burnout, along with their reported effectiveness. The specific research question was as follows: What interventions have been used to reduce lecturers’ burnout, and how effective have they been reported to be? The review seeks to pinpoint existing knowledge gaps regarding burnout interventions for lecturers and highlight potential opportunities for future research.

## 3. Materials and Methods

This study employed the Systematic Literature Review (SLR) methodology to ensure the reproducibility of research findings for future studies. A comprehensive literature review was conducted to address the research question, involving the retrieval and assessment of relevant data from multiple academic databases. This study’s main analytical method was a systematic literature review, which entailed looking through and assessing relevant data from five databases (PROSEPRO) in February 2025: CRD420250649817.

### 3.1. Search Strategy

To ensure comprehensive and reproducible identification of relevant studies, we conducted a comprehensive search in spring 2025 across five electronic databases: Web of Science (WOS), APA PsycINFO, Scopus, PubMed, and ERIC. This review focused on empirical studies that investigated interventions to mitigate burnout among university lecturers, published between January 2020 and December 2024.

The development of the search strings was guided by the parameters of the study, including the research topic (burnout and intervention), the type of studies (empirical), and the target participants (university lecturers). These initial parameters were refined into official inclusion criteria for the studies ultimately selected for review.

Keywords were selected through consultation with a subject librarian and two domain experts in psychology. The keywords were successfully combined using the Boolean operators (AND, OR). A variety of keywords were employed, including (1) burnout, burn-out, burned out; (2) lecturers, university instructors, college instructors; and (3) intervention, therapy, management, treatment, interventions, strategies, techniques. These were combined using Boolean operators: (burnout OR burn-out OR burned out) AND (lecturer* OR university instructor* OR college instructor*) AND (intervention OR treatment OR therapy OR strategy OR management OR technique). Search strategies were tailored to each database to ensure optimal sensitivity and specificity, with keyword structures customized as summarized in Table 1. The initial search yielded 437 articles across the five databases.

Two independent reviewers (Reviewer A and Reviewer B) conducted title and abstract screening using Rayyan software (Version 1.6.0). Full-text articles were assessed against inclusion criteria. Discrepancies were resolved through consensus, and a third reviewer was consulted when necessary. To ensure the rigor of the study selection process, inter-rater reliability was assessed using Cohen’s Kappa. The agreement between the two reviewers during full-text screening was κ = 0.82, indicating substantial agreement (Landis & Koch, 1977). This level of reliability supports the robustness and transparency of the review process.

### 3.2. Inclusion/Exclusion Criteria for Studies

Inclusion criteria and exclusion criteria were established to provide a comprehensive understanding of interventions aimed at reducing burnout among university lecturers, based on the research objectives. The SPIDER and PICO frameworks were primarily employed to identify relevant studies in this review. These tools enable a comprehensive and efficient retrieval of relevant literature. Specifically, PICO focuses on Population, Intervention, Comparison, and Outcome, while SPIDER emphasizes Sample, Phenomenon of Interest, Design, Evaluation, and Research type (Cooke et al., 2012; Eriksen & Frandsen, 2018). Both frameworks were employed in the present study to identify systematic reviews and meta-analyses by applying the predefined inclusion and exclusion criteria. A detailed list of these criteria is provided in Table 2.

The inclusion criteria were as follows: (1) The years of publication are 2020–2024; (2) inclusion of the keywords “burnout”, “intervention”, and “lecturers”; (3) articles published in English; and (4) studies employing quantitative, qualitative, or mixed methods to offer a comprehensive perspective on the research topic. The decision to focus on the 2020–2024 timeframe was influenced by several factors. First, this period saw the widespread use of online resources, enabling the examination of interventions aimed at reducing burnout among college lecturers. Second, focusing on these years ensures access to up-to-date and relevant literature, capturing the most recent developments and trends in the field’s understanding. Finally, limiting the time period makes it easier to conduct a more comprehensive literature review, which improves our comprehension of university lecturers’ burnout.

The excluded criteria were as follows: (1) Articles that concentrate on people who are not college lecturers, such as professors, students, primary and secondary school teachers, and principles, will not be reviewed; (2) Studies unrelated to interventions to alleviate lecturers’ burnout will not be included; (3) Articles that fail to directly answer the research question will be excluded. (4) Non-empirical articles such as opinion pieces, editorials, theoretical papers, and literature reviews will not be subject to the review process; (5) Articles published in languages other than English will be excluded, as the analysis is limited to English-language publications; (6) Studies for which the full text cannot be accessed will be excluded.

### 3.3. Data Extraction

**Figure 1:**
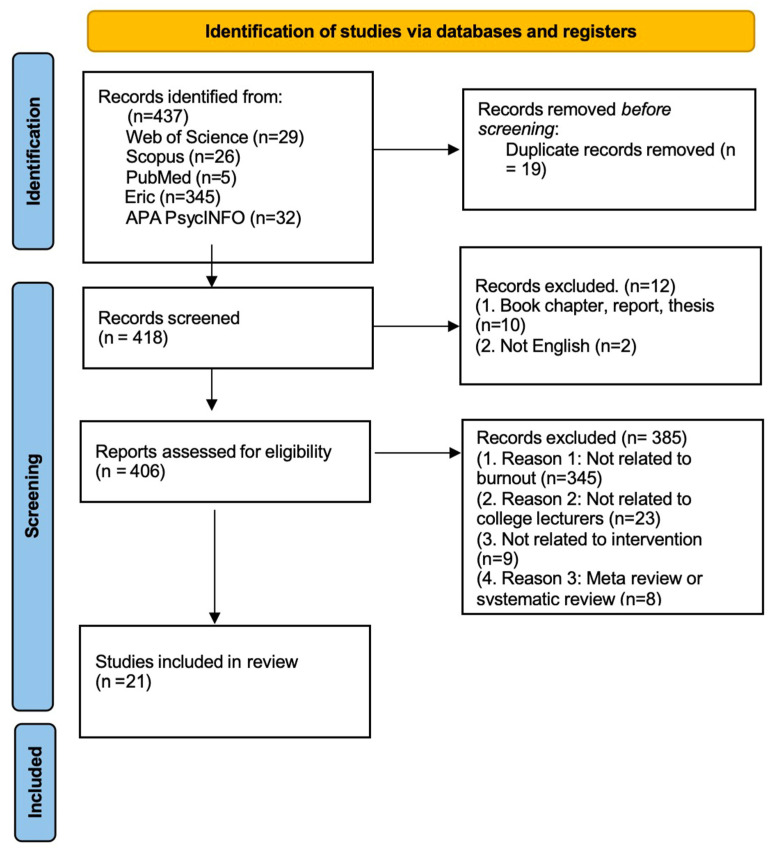
PRISMA Flowchart depicting the inclusion and exclusion process of articles at each stage of the systematic review.

Firstly, 437 manuscripts were initially identified, but 19 duplicated manuscripts were removed, bringing the total number of records to 418 for further screening. A total of 12 reports were excluded from detailed review for various reasons, such as being reviews, conference papers, book chapters, or magazine articles (10 records), or not being written in English (2 records). Following this, a rigorous screening process was conducted to evaluate the relevance of titles, abstracts, and keywords to the research objectives, leading to the exclusion of a substantial number of records. Among the retrieved articles, 385 were removed for the following reasons: 345 were not related to burnout, 23 did not focus on college lecturers, 9 were unrelated to interventions, and 8 were review articles. Ultimately, 21 articles were chosen for further coding and analysis. The PRISMA flowchart (Figure 1) illustrates the number of articles included and excluded at each stage of the review process. EndNote X9 was utilized to organize and format the bibliography, while Microsoft Word (2019) and Excel (2021) were employed for data analysis and management.

### 3.4. Quality Assessment

The quality of the studies included in this review was evaluated using Crowe’s Critical Appraisal Tool (CCAT), which is particularly suitable for assessing diverse research designs, such as mixed-methods and quantitative and qualitative studies. This flexibility is essential given the variety of methodologies examined. CCAT also boasts high reliability. It comprises eight sections: Introduction, Background, Methods, Sampling, Data Collection, Ethics, Results, and Discussion. Each category is rated on a five-point scale, with a maximum possible score of 40. Detailed guidance for scoring is provided in the CCAT User Guide. Two independent reviewers will conduct the quality assessment, and any disagreements will be resolved through discussion and consensus. Based on the CCAT scores, studies will be classified into three quality tiers: low quality (score < 25), medium quality (score 25–30), and high quality (score > 30). CCAT prioritizes the detailed evaluation and documentation of results for each category, rather than relying solely on the overall score. This ensures that studies excelling in certain areas but lacking in others are not inappropriately ranked above those demonstrating consistent quality across all categories. The CCAT scores and detailed attributes for the 21 studies included in this review are provided in Table A1.

### 3.5. Synthesis and Analysis of Results

In the coding stage, we systematically gathered and structured data on interventions, burnout, and university lecturers. Each article was categorized based on several factors, including the author, publication year, country, title, research objectives, methods, participants, and results. Through inductive analysis, a descriptive code was assigned to each article and its corresponding subcategory. For the analysis of qualitative data, thematic analysis was the main technique employed. This approach helped to summarize and categorize the articles before developing a framework that ensured alignment between the data and the framework. This approach was used to code, classify, and refine themes that emerged from the raw data. The current study follows the six-step model proposed by Clarke and Braun, which includes the subsequent steps: (1) have a thorough understanding of the data; (2) create codes; (3) recognize themes; (4) review themes; (5) define themes; and (6) interpret themes (Clarke & Braun, 2017; Braun & Clarke, 2019).

We began the thematic analysis by deeply engaging with the data to gain a comprehensive understanding of the articles relevant to the research question. Using an inductive approach, we assigned codes to the articles, enabling themes to naturally arise from specific observations in the empirical studies. This involved examining key aspects such as the author, publication year, country, study objectives, research design, and conclusions, while also noting recurring subject terms. Next, we reviewed all identified themes to remove overlaps and merge closely related ones. In the fifth step, we consolidated and defined the shortlisted themes, refining them iteratively until all sub-themes were logically organized under their main themes. Finally, we further polished and defined the themes, with the first two authors reaching agreement on each theme through thorough discussion and evaluation of its connection to the research question. Disagreements were resolved by consulting a third author to maintain objectivity. After validating the initial codes, we refined the themes, and the final set, along with their analysis, is presented in the following section. By putting preliminary codes into broader categories and participating in author discussions, two primary themes were identified: (1) the interventions used to reduce lecturers’ burnout, and (2) the reported effectiveness of these interventions.

## 4. Results

### 4.1. Contexts and Characteristics of the Studies

**Figure 2:**
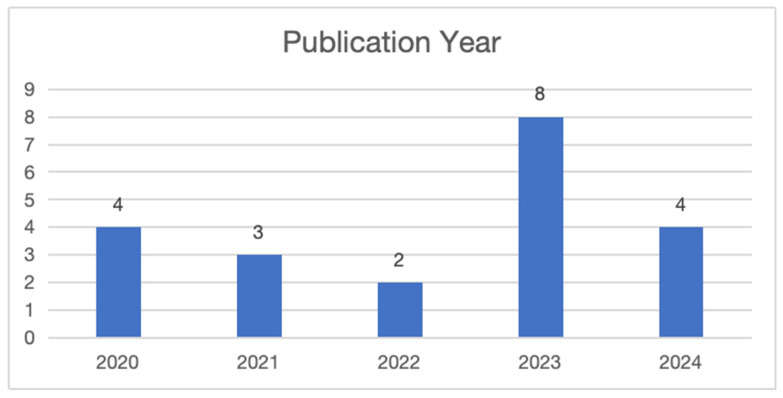
Publications arranged by publication year.

**Figure 3:**
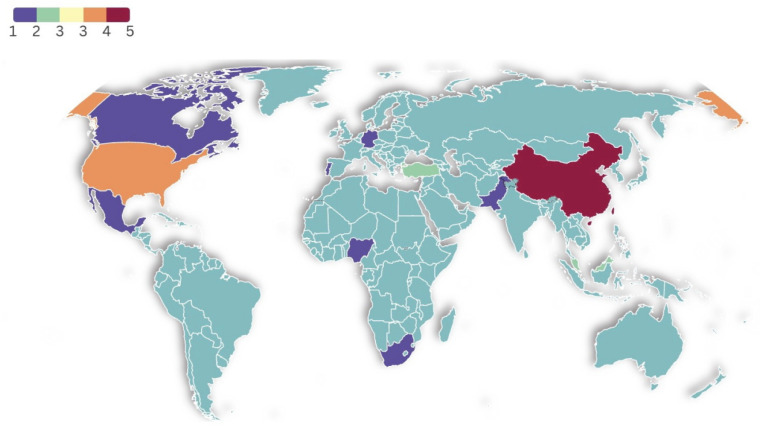
Geographical distribution of journal articles.

**Figure 4:**
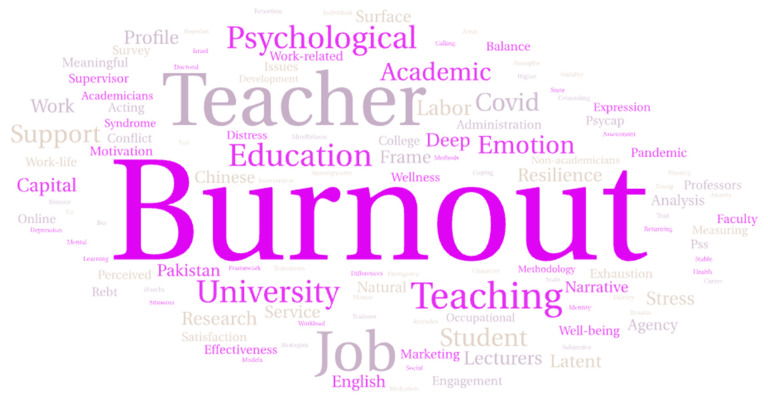
Word cloud map generated in 21 documents.

As shown in Figure 2, a systematic review was conducted using 21 selected articles published between 2020 and 2024. The data highlight the number of publications per year, starting with four publications in 2020, followed by three in 2021, and two in 2022. The number peaked in 2023 with eight publications, reflecting a significant increase in research focused on interventions to reduce burnout among university lecturers. In 2024, there were four publications, indicating that interest in this topic remains strong. Figure 3 summarizes the contributions from various countries to the research on interventions for burnout among university lecturers. It shows that China and the United States are the leading contributors, with China publishing five articles and the United States four. Other notable contributors include Turkey and Malaysia, each with two publications, followed by Pakistan, South Africa, Portugal, Germany, Canada, Mexico, Nigeria, and Israel, each with one publication. Most studies in the review employed a quantitative research design, while a article utilized a qualitative research design. The word cloud analysis revealed that the term “burnout” was mentioned most frequently, appearing 23 times, followed by “teacher” (12 mentions), “job” (9), “teaching” (6), and “education” (5) (See Figure 4).

### 4.2. Interventions to Reduce Burnout Among Lecturers

The burnout experienced by university lecturers may be shaped by their unique professional positioning. In many cases, lecturers are neither tenured faculty with strong institutional backing, nor adjuncts with clearly defined, albeit limited, responsibilities. This ambiguous status can lead to feelings of marginalization and reduced professional identity. Compared to adjunct faculty, lecturers often carry heavier teaching loads. Yet unlike full-time tenured staff, they frequently lack the institutional authority, resources, and long-term job security that might buffer against occupational stress. While some lecturers may be assigned administrative or service roles, this varies widely across institutions and should not be assumed as a universal duty. The inconsistent expectations placed on lecturers can further amplify role conflict, emotional exhaustion, and depersonalization.

In response to these challenges, this systematic literature review identified a range of interventions aimed at alleviating burnout among university lecturers. A total of 21 articles were reviewed, which discussed seven distinct types of interventions to address this issue. Among these, the role of social support emerged as a particularly robust and recurrent theme.

#### 4.2.1. Utilizing Social Support to Combat Burnout

Research consistently emphasizes that social support plays a pivotal role in mitigating the adverse psychological effects of burnout among university lecturers. Across various studies, social support is shown to help individuals manage stress, enhance emotional well-being, and maintain a sense of professional and personal stability.

##### The Role of Social Support Networks

The studies by Heng et al. (2020), Li et al. (2020), Mohamed et al. (2021), Hoter and Reiner (2023), and Cicco (2024) highlight the protective nature of strong social support networks in buffering the burnout caused by the demands of academic work. These networks, including colleagues, family, and institutional connections, provide essential emotional reassurance, practical assistance, and a sense of belonging. These resources are instrumental in helping lecturers cope with burnout and sustain their psychological health.

However, the effectiveness of such networks is not uniform across contexts. The availability and quality of social support can vary significantly between institutions and geographic regions. For instance, in environments characterized by collegial collaboration and accessible institutional resources, lecturers may find meaningful support. Conversely, in more hierarchical or isolated academic settings, social support may be minimal or absent, exacerbating feelings of stress and professional alienation. Moreover, while emotional support is undeniably important, it often addresses only the symptoms—rather than the root causes—of burnout. Issues such as excessive workload, limited professional autonomy, and unclear job expectations are structural in nature. Thus, while emotional reassurance from colleagues and family may offer short-term relief, it cannot be a substitute for comprehensive, systemic reform. Social support should be viewed as one component within a broader suite of institutional strategies designed to reduce burnout.

##### Institutional Support and Autonomy

Organizational support is equally essential in addressing the root causes of burnout. Tan and Kim (2024) emphasize the importance of organizational support in helping lecturers retain their professional autonomy and master their academic goals. Organizational practices that promote autonomy and provide necessary resources for professional development are critical in preventing burnout and fostering long-term well-being. Genuine concern and appreciation from institutional leaders, along with accessible wellness opportunities, are also essential in combating burnout. Institutions of higher education should allocate resources toward faculty wellness programs to reduce burnout (Koster & McHenry, 2023). These elements are vital in ensuring that lecturers can effectively navigate the challenges of their roles without feeling overwhelmed or disengaged. However, several institutional barriers may hinder the implementation of meaningful support. Many universities face pressures that limit faculty autonomy, including rigid curricular structures, performance metrics, and increased administrative demands. As a result, even well-intentioned support programs may fail to address burnout unless universities also commit to reducing bureaucratic constraints and enabling flexible, self-directed work.

##### Support from Family and Colleagues

Malik and Björkqvist (2021) highlight the essential role that family members and colleagues play in reducing the negative consequences of burnout. Furthermore, recognition and rewards from colleagues and department heads are vital in maintaining lecturers’ morale and alleviating stress. Hoter and Reiner (2023) further argue that administration and faculty leaders should focus on enhancing the emotional well-being of staff members by recognizing and strengthening the support systems available to them, including family and friends. However, reliance on personal networks can pose its own challenges. The repeated emotional labor of supporting a stressed lecturer may lead to fatigue or strain in family relationships and workplace dynamics. In some cases, this burden may even become a secondary source of stress for the supporters themselves.

#### 4.2.2. Training Programs to Mitigate Burnout

Recent research highlights that targeted training programs can play a critical role in preventing and mitigating university lecturers’ burnout. Five studies have explored the important role of training programs in alleviating burnout among university lecturers. This section explores the effectiveness of early training programs, professional development, trauma-informed and mindfulness-based interventions, and support training for stakeholders in preventing lecturers’ burnout.

##### Early Training and Professional Development

The implementation of early training programs, such as those proposed by Carstensen et al. (2024), provides a promising strategy for preventing burnout. By offering training before lecturers begin their full-time teaching roles, these programs aim to equip new educators with the necessary skills and coping mechanisms to handle the challenges they will face in the classroom. Such early interventions may be particularly valuable in alleviating the emotional exhaustion commonly experienced during the first year of teaching, when individuals are often most vulnerable to burnout. However, while early training interventions can provide initial relief, the broader question remains as to how effectively these programs can address the evolving demands and stressors that lecturers encounter throughout their careers. Emotional exhaustion tends to accumulate over time, especially for those who continue to face high teaching loads, limited resources, and the increasing pressures of academic accountability. Consequently, early training, though beneficial, may be insufficient without ongoing support and professional development opportunities throughout lecturers’ careers.

Castro et al. (2023) emphasize the importance of professional development programs, including educational technology training and other relevant skill-building initiatives, in preventing emotional exhaustion. This approach is further supported by research from Hoter and Reiner (2023), who suggest that engaging in professional development activities not only exposes lecturers to new challenges but also provides opportunities to connect with new colleagues, which can significantly aid in alleviating burnout. However, the effectiveness of professional development depends largely on how well these programs are designed and integrated into the lecturers’ existing responsibilities. If professional development activities are seen as additional burdens rather than valuable tools for improvement, they may exacerbate rather than alleviate stress.

##### Mindfulness-Based and Trauma-Informed Interventions

Mindfulness-based and trauma-informed interventions have been increasingly recognized as effective strategies for reducing burnout. Kim et al. (2021) proposes that integrating trauma-sensitive approaches with mindfulness-based Social-Emotional Learning (SEL) interventions can help educators develop resilience against workplace stressors. These approaches encourage lecturers to adopt trauma-sensitive attitudes, which can alleviate emotional strain and enhance psychological well-being. While mindfulness and trauma-informed interventions are powerful tools for emotional regulation, they do not directly address the structural sources of burnout, such as workload imbalance, job insecurity, and lack of institutional support. To be truly effective, mindfulness and trauma-informed approaches must be integrated into broader institutional reforms that reduce systemic stressors and support lecturers in their roles.

##### Stakeholder Training for a Supportive Environment

Support can be extended by training not only teachers but also other stakeholders like parents, school administrators, and policymakers. By offering targeted programs for these groups, institutions can create a more supportive environment to help reduce burnout. As Sözer-Boz et al. (2024) note, pre-service teacher education should include courses focused on resilience and communication with students and parents. These courses can equip new teachers to better manage work–life balance and emotional challenges, ultimately enhancing their well-being and preparing them for the demands of the profession. While stakeholder training holds promise, it introduces the complexity of intergroup dynamics. The success of these programs depends not only on the quality of the training but also on how effectively the various stakeholders collaborate and communicate.

#### 4.2.3. Cultivating Supportive Work Environments to Mitigate Burnout

A growing body of research underscores the importance of creating wellness cultures and fostering supportive work environments as critical strategies for reducing burnout. This section examines the role of institutional wellness cultures, effective communication, and interpersonal relationships in mitigating burnout and promoting faculty well-being.

##### Establishing Wellness-Oriented Academic Environments

Creating a wellness-focused institutional culture is a fundamental strategy for preventing burnout. Melnyk et al. (2023) emphasize that academic leaders must prioritize faculty well-being by implementing evidence-based interventions and ensuring access to necessary resources and support systems. Inclusive wellness initiatives can help mitigate stress and enhance faculty members’ mental health. However, the success of such initiatives depends heavily on institutional follow-through and the depth of integration into academic culture. Superficial or performative wellness campaigns (e.g., sporadic mental health workshops without addressing excessive workloads or unclear job expectations) can create a disconnect between institutional rhetoric and the lived realities of faculty. This gap may even increase cynicism and disillusionment, exacerbating burnout rather than alleviating it.

##### Strengthening Communication and Workplace Relationships

Effective communication and strong working relationships between teachers and administrators are crucial for reducing burnout. Malik and Björkqvist (2021) highlight the importance of open communication, performance feedback, and social activities, such as informal coffee breaks, lunch gatherings, or dinners, which can foster camaraderie among faculty members. Improving communication and knowledge sharing between colleagues and department heads is essential for building a supportive environment, which can significantly reduce feelings of isolation and stress. Yet, cultivating such communication-rich environments requires a shift from hierarchical, bureaucratic norms to more participatory and empathetic forms of governance. Many universities operate under rigid administrative structures where communication tends to be top-down, formal, and impersonal.

##### Promoting Harmonious Interpersonal Relationships

In addition to organizational strategies, the importance of maintaining harmonious interpersonal relationships cannot be overstated. Sözer-Boz et al. (2024) argue that building and managing relationships with students and superiors is a primary source of both motivation and burnout for teachers. By encouraging respectful and collaborative interactions, institutions can reduce the emotional strain on lecturers, which often contributes to burnout. However, this ideal is often undermined by competitive institutional cultures, ambiguous role expectations, or misaligned power dynamics.

#### 4.2.4. REBT Intervention to Mitigate Burnout

Research has shown that Rational Emotive Behavior Therapy (REBT) is effective in reducing symptoms of job burnout among lecturers. Eseadi et al. (2023) advocate for the integration of online REBT programs into university mental health policies to address burnout symptoms and enhance faculty well-being. However, while REBT can address internalized stress responses, it does not inherently alter the external structures that produce burnout.

#### 4.2.5. Managing Work Demands and Teaching Transitions

Transparent workload models and strategic staffing plans ensure equitable work distribution and prevent excessive burdens on academic staff (Fynn & Walt, 2023). Additionally, reassessing work demands, including research expectations and Key Performance Indicators (KPIs), can help create more sustainable work conditions. Kassim et al. (2020) suggest that universities implement regular screening and surveillance systems, such as online surveys, to monitor faculty burnout and provide timely interventions. While these measures can prevent overburdening staff, their success depends heavily on institutional commitment to transparency and enforcement.

#### 4.2.6. Enhancing Psychological Resilience and Character Strengths

Psychological capital, comprising self-efficacy, optimism, hope, and resilience, is a vital personal resource in preventing burnout and supporting the mental health of college teachers (Yin, 2023). Developing resilience enables faculty members to better manage stress, recover from setbacks, and maintain motivation. Resilience, a key component of psychological capital, has been identified as a crucial factor in protecting teachers from burnout. García-Rivera et al. (2022) emphasize that resilience serves as a protective factor against burnout by helping educators navigate workplace challenges. Furthermore, identifying and utilizing character strengths through mindfulness interventions and professional development programs can enhance faculty engagement and reduce burnout (Lian et al., 2021).

#### 4.2.7. Promoting Work–Life Balance and Flexible Work Conditions

Maintaining a healthy work–life balance is crucial in preventing burnout. Taylor and Frechette (2022) recommend that instructors use interactive platforms, peer collaboration, and digital resources to manage student interactions effectively without compromising their well-being. Additionally, flexible work arrangements, such as remote teaching options and adaptable schedules, have been shown to alleviate stress and improve faculty work–life balance (Yildirim & Senel, 2023). By providing such options, universities can create a more sustainable and supportive academic environment. Yet, flexibility is not a panacea. Without clear boundaries and institutional safeguards, remote work can blur work–life lines, making it difficult for lecturers to disconnect. Furthermore, flexible policies must be equitably available—if only certain departments or roles benefit from flexibility, this can exacerbate existing inequalities and fuel further resentment or stress.

## 5. Discussion

Burnout is a significant global issue, and identifying effective interventions to alleviate or reduce burnout among lecturers is a crucial step in addressing this challenge. This systematic review analyzed 21 studies focused on interventions aimed at mitigating or preventing burnout, including social support, training programs, supportive work environments, Rational Emotive Behavior Therapy (REBT), workload management, psychological resilience, and work–life balance. The following discussion expands on the implications, limitations, and directions for future research and institutional policies.

Social support is a promising approach and a widely used method for reducing burnout and improving the emotional well-being of university lecturers. The Conservation of Resources (COR) theory provides a valuable framework for understanding this relationship. According to Hobfoll et al. (2002), people strive to gain and preserve resources, and they experience burnout when those resources are actually lost or do not increase as planned. Social support can be a crucial resource, helping individuals cope with burnout by offering the emotional, informational, or material assistance needed to navigate difficult circumstances. These findings align with previous studies, which have shown that perceived social support significantly correlates with reduced burnout. Lecturers who experience strong social support, from both colleagues and personal networks such as family and friends, report better coping abilities, lower stress, and improved resilience (Taylor & Frechette, 2022; Kolomitro et al., 2020). However, the effectiveness of social support varies depending on institutional policies, cultural contexts, and individual coping styles. While it serves as a protective buffer, poorly matched or overly intrusive support can undermine autonomy and exacerbate stress, particularly in environments where academic freedom is central. Furthermore, an over-reliance on external support may limit the development of internal coping strategies, leading to potential dependency or learned helplessness. Future research should investigate the role of institutional and cultural contexts in shaping the outcomes of social support interventions, exploring how different types of support (e.g., emotional vs. informational) can be optimized for diverse academic settings.

Training programs, particularly those focused on mindfulness, trauma-informed approaches, and professional development, have shown promise in preventing and reducing burnout. Research has also highlighted the positive impact of professional coaching, which has been shown to reduce emotional exhaustion and impostor syndrome while increasing self-compassion (Fainstad et al., 2022). Additionally, studies demonstrate that Mindfulness-Based Interventions (MBIs) are effective in alleviating burnout among lecturers (Carroll et al., 2021; X. Cheng et al., 2021; Czerwinski et al., 2021; de Carvalho et al., 2021). However, the effectiveness of training is not guaranteed, especially when programs are generic or do not address the specific stressors of the academic environment. In particular, a one-size-fits-all approach may fail to resonate with lecturers, particularly in institutions that perpetuate systemic issues such as a “publish-or-perish” culture or precarious employment conditions. Moreover, training should not be viewed solely as an individual-level intervention but as part of a broader organizational transformation. Without parallel reforms in workload management, faculty recognition systems, and administrative support, training programs risk becoming mere band-aid solutions. Future research should focus on evaluating the impact of customized training programs that are contextually relevant to different types of institutions and academic disciplines.

Fostering supportive work environments is also a critical strategy for mitigating burnout among university lecturers. By prioritizing wellness, improving communication, and promoting positive interpersonal relationships, academic institutions can create a work culture that supports faculty well-being and reduces stress. These efforts not only help prevent burnout but also contribute to a more engaged, resilient, and satisfied faculty, ultimately enhancing the overall effectiveness of academic institutions. This aligns with the findings of Hammoudi Halat et al. (2023) and Melnyk et al. (2023), who emphasized that creating a fostering supportive work environments was an effective strategy for reducing burnout in teachers. However, a supportive work environment must be accompanied by tangible changes to workload distribution, administrative practices, and resource allocation. Simply fostering a positive atmosphere without addressing systemic issues such as overwork or lack of resources can inadvertently worsen burnout. Faculty members may feel unsupported if their workloads are excessive or if institutional policies fail to address the root causes of stress. Future research should explore the intersection of work environment factors and workload management, examining how policies that balance the demands placed on faculty can be more effectively integrated into institutional culture.

Innovative interventions, such as Rational Emotive Behavior Therapy (REBT), managing work demands, enhancing psychological resilience, fostering character strengths, and promoting work–life balance and flexible work conditions, can significantly mitigate burnout among university lecturers. By fostering supportive workplace policies, ensuring equitable work distribution, and encouraging self-awareness of strengths, universities can create a healthier academic environment. These proactive measures not only reduce burnout but also enhance lecturers’ well-being, resilience, and professional satisfaction. Among these strategies, REBT and character strength development have proven to be particularly effective in preventing and alleviating burnout. Consistent with previous research, REBT has demonstrated significant positive effects on reducing work-related stress and burnout, particularly among special educators (Onuigbo et al., 2018; Ogba et al., 2020; Okeke et al., 2021). Furthermore, identifying and leveraging lecturers’ personality strengths presents a promising approach to reducing burnout prevalence and mitigating its negative consequences (Roloff et al., 2022).

Maintaining work–life balance has proven to be an effective strategy for reducing lecturers’ burnout, a finding that is supported by the findings of Ogakwu et al. (2022) and Hammoudi Halat et al. (2023), who found that enhancing work–life balance management can significantly mitigate burnout among lecturers. These findings are in line with the findings of Xie et al. (2022), who found that psychological capital has been identified as a crucial resource in reducing the occurrence of lecturer burnout. However, while these interventions have demonstrated efficacy, they must be implemented within the context of organizational change. The success of such programs relies heavily on systemic support, including equitable work distribution, supportive leadership, and a commitment to reducing excessive demands.

## 6. Conclusions

Burnout within the teaching profession is a pervasive problem, underscoring the importance of effective interventions. This review synthesizes and examines a range of strategies that have been employed to address and reduce burnout among lecturers. By presenting this evidence, the review aims to inform both lecturers and universities in developing policies and adopting programs that effectively address burnout. Several key interventions were identified, including utilizing social support, training programs, fostering supportive work environments, Rational Emotive Behavior Therapy (REBT), managing work demands and teaching transitions, enhancing psychological capital and character strengths, and promoting work–life balance and flexible work conditions. These strategies collectively offer a comprehensive approach to preventing and alleviating burnout among lectures, but their success is contingent on the organizational and cultural contexts in which they are implemented. To optimize the impact of these interventions, universities must engage in systemic reforms that address workload, recognition, and support structures. Future research should continue to explore how different interventions interact within specific institutional contexts and identify best practices for tailoring strategies to diverse academic environments. By doing so, academic institutions can better support their faculty members, mitigate burnout, and enhance the overall well-being of university lecturers.

However, this systematic literature review has several limitations that may affect the breadth and depth of its conclusions. It focuses solely on empirical, peer-reviewed journal articles, excluding theoretical papers, books, and grey literature such as conference proceedings and book chapters, which may provide valuable insights. The restriction to English-language publications also introduces language bias, potentially overlooking relevant studies from non-English-speaking regions. Additionally, most of the included studies originate from Western contexts—particularly the U.S. and Europe—limiting the applicability of findings to non-Western academic settings, where cultural and institutional differences may affect both burnout and intervention outcomes. Methodological variability further complicates comparisons across studies. Many relied on self-reported measures prone to social desirability and recall bias, and the predominance of cross-sectional designs hinders causal inferences. Finally, the use of descriptive and thematic analysis rather than meta-analytic techniques limits the ability to statistically assess the overall effectiveness of interventions.

While some interventions—particularly those aimed at fostering supportive work environments—pose implementation challenges; universities must prioritize and actively implement strategies specifically aimed at reducing and preventing burnout among lecturers. Implementing appropriate, school-based interventions across institutions is essential for enhancing lecturers’ ability to cope with job-related stress. Current research primarily focuses on individual-level strategies for mitigating burnout, but future studies should place greater emphasis on organizational and institutional approaches. These may include reducing workload-related stress, improving welfare benefits, and fostering a more harmonious and supportive work environment.

Despite methodological differences and variations in the interventions reviewed, each approach demonstrated effectiveness in reducing lecturer burnout. However, due to inconsistencies in study methodologies and the quality of the included research, it is not possible to determine which intervention is the most effective in supporting educators’ mental health. Future research should aim to bridge these gaps by employing standardized methodologies and expanding the scope of investigation to develop more comprehensive and evidence-based solutions for addressing lecturer burnout.

## Figures and Tables

**Table 1:** Search Strings.

Search Query	Keywords in Title, Abstract, and Keyword
1	“burnout” OR “burn-out” OR “burned out”
2	“lecturers” OR “university instructors” OR “college instructors”
3	“intervention” OR “therapy” OR “management” OR “treatment” OR “interventions” OR “strategies” OR “techniques”
Final	1 + 2 + 3

Databases: Web of Science, Scopus, APA PsycINFO, ERIC, PubMed. Publication Dates: 1 January 2020 to 31 December 2024.

**Table 2:** Inclusion and Exclusion Criteria.

Criteria	Inclusion	Exclusion
Population	College lecturers	Primary and secondary school teachers, professors, students, principles
Interventions	None	None
Comparisons	None	None
Country setting	Any country	None
Outcomes	Studies related to interventions to reduce burnout	Studies not related to interventions to relieve lecturers’ burnout
Setting	Studies with broad definitions of interventions and burnout	Studies not related to interventions to reduce burnout
Language	English or translated into English.	Not in English.
Study types and designs	Quantitative research Qualitative research Mixed research	Studies without empirical data.

## Data Availability

Not applicable.

## Appendix A

**Table A1:** The Characteristics and CCAT Scores of the 21 Studies.

No.	Authors, Year	Country	Title	Aim(s)	Participant Characteristics	Study Type	Research Design	Main Findings	CCAT Scores
1	(Malik & Björkqvist, 2021)	Pakistan	An Evidence-Based Framework for Reducing Occupational Stress and Burnout in Pakistani Universities	Design a framework for university administrations to provide guidelines for the effective management of the issues of occupational stress and burnout	320 university teachers	A cross-sectional study	Quantitative research	Professional development activities related to creating awareness about occupational stress, burnout, and stress management skills should be encouraged	37
2	(Koster & McHenry, 2023)	USA	Areas of work–life that contribute to burnout among higher education health science faculty and perception of institutional support	Identify how a health science faculty at one institution perceived challenges related to the COVID-19 pandemic in their role and to glean opportunities for institutions to increase the degree of support for faculty.	39 participants	A cross-sectional study	Quantitative research	Flexibility in workload, genuine concern and appreciation expressed by institutional leaders, and accessible wellness opportunities may help to offset these negative feelings.	38
3	(Mohamed et al., 2021)	Malaysia	Burnout and its relationship to psychological distress and job satisfaction among academicians and non-academicians in Malaysia	Ascertain the prevalence of burnout and its associated risk factors among university staff, involving both academicians and non-academicians and relate these to their job satisfaction	411 teachers	A cross-sectional study	Quantitative research	Academicians suffer from high levels of burnout in aspects of personal, work, and client-related matters, and this has contributed to higher psychological distress among them and significantly affect their job satisfaction.	38
4	(Fynn & Walt, 2023)	South Africa	Examining staff burnout during the transition to teaching online due to COVID-19 implications	Assess the prevalence and severity of burnout symptoms among academics and its impact on work engagement	147 lecturers	A cross-sectional study	Quantitative research	Teaching transitions are beneficial for preventing and alleviating burnout among university lecturers.	36
5	(Tan & Kim, 2024)	China	Exploring the relationship between teacher motivation and teacher burnout among Chinese college English teachers	Explore the relationship between teacher motivation and burnout, as well as the factors that affect teacher burnout among Chinese college English teachers.	261 college English teachers	A cross-sectional study	Quantitative research	Organizational support needs to be provided for teachers to maintain their mastery goal orientations and increase their autonomy as professional teachers.	37
6	(Castro et al., 2023)	Portugal	Burnout, resilience, and subjective well-being among Portuguese lecturers’ during the COVID-19 pandemic	Describe burnout amongst lecturers working in Portugal and analyze potential determinants of burnout during the COVID-19 pandemic.	331 lecturers	A cross-sectional study	Quantitative research	Support strategies such as educational technology training, professional development programs, emotional support resources, and workload flexibility are useful for enhancing lecturers’ resilience and overall life satisfaction and could potentially help mitigate burnout and improve the well-being of educators	39
7	(Carstensen et al., 2024)	Germany	How stable is student teachers’ emotional exhaustion? Disentangling different components of stability and change using the STARTS model	Gain a deeper understanding of the genesis, stability, and trajectories of change of emotional exhaustion	Sample 1: N = 4510; Sample 2: N = 2034	A cross-sectional study	Quantitative research	Intervention measures should be employed at an early stage to mitigate the negative consequences of burnout in subsequent career steps.	36
8	(Kim et al., 2021)	Canada	Impact of Trauma-Informed Training and Mindfulness-Based Social–Emotional Learning Program on Teacher Attitudes and Burnout: A Mixed-Methods Study	Investigate the benefits of trauma-informed training and Mind UP delivery on educator attitudes and burnout.	112 educators	A cross-sectional study	Quantitative research	Infusing trauma-informed training with an existing mindfulness-based SEL intervention may encourage teachers to embrace trauma-sensitive attitudes and reduce burnout.	39
9	(García-Rivera et al., 2022)	Mexico	Influence of Resilience on Burnout Syndrome of Faculty Professors	Describe the relationship between resilience and burnout when facing the COVID-19 pandemic	831 lecturers and professors	A cross-sectional study	Quantitative research	Supervisors should begin paying attention not only to the purely theoretical aspect of teaching enabling but also to making a greater effort to connect with professors and lecturers, giving weight and attention to their difficulties, and fostering a classroom climate more devoted to sharing the problematic aspects of online teaching.	38
10	(Eseadi et al., 2023)	Nigeria	Intervention for job burnout reduction among a sample of Nigerian lecturers	Nigerian university history lecturers were examined in respect of job burnout prior to and after an online psychological intervention that followed the Rational Emotive Behavior Therapy (REBT) principles and practices.	80 university history lecturers	A cross-sectional study	Quantitative research	University history lecturers can benefit from online psychological intervention that targets job burnout reduction.	39
11	(Yildirim & Senel, 2023)	Turkey	The Relationship Between Work–Life Balance and Academic Burnout Levels of Academic Staff	Determine the relationship between the perceptions of burnout and the work–life balance of academic staff	352 academic staff.	A cross-sectional study	Quantitative research	Flexible working conditions that can balance work and life conditions, the distance between home and workplace in terms of transportation, and provide a more favorable environment for academic studies.	38
12	(Melnyk et al., 2023)	USA	The state of mental health, burnout, mattering and perceived wellness culture in Doctoral prepared nursing faculty with implications for action	(1) Describe the current rate of depression, anxiety, and burnout in PhD- and DNP-prepared nursing faculties and tenure and clinical faculties across the United States; (2) determine if differences exist in mental health outcomes between PhD- and DNP-prepared faculties and tenure and clinical faculties; (3) explore whether or not wellness culture and mattering to the organization influence faculty mental health outcomes	224 faculty	A cross-sectional study	Quantitative research	Academic organizations need to build wellness cultures and provide infrastructures that offer evidence-based interventions to support faculty well-being.	36
13	(Yin, 2023)	China	Psychological capital moderates the effect of emotional labor strategies on job burnout in college teachers	Explore the correlations of psychological capital, job burnout, and emotional labor strategies.	434 teachers	A cross-sectional study	Quantitative research	For teachers with high psychological capital, expression of natural emotion and deep acting were both significantly correlated with job burnout, but the correlation between surface acting and burnout was not significant; however, for teachers with low psychological capital, surface acting and burnout were significantly correlated, but the correlations between expression of natural emotion and burnout and between deep acting and burnout were not significant.	39
14	(Heng et al., 2020)	China	The mechanism of teaching–research conflict influencing job burnout among university teachers: The roles of perceived supervisor support and psychological capital	Examine the relationship between teaching–research conflict and job burnout among university teachers and the moderating role of Perceived Supervisor Support (PSS) and Psychological Capital (PsyCap) in this relationship.	488 university lecturers	A cross-sectional study	Quantitative research	Teaching–research conflict was positively linked to emotional exhaustion and depersonalization but negatively linked to personal accomplishment; PSS moderated the effects of teaching–research conflict on both emotional exhaustion and depersonalization but did not act as a moderator in the relationship between teaching–research conflict and personal accomplishment; PsyCap moderated the effect of teaching–research conflict on all three dimensions of job burnout.	38
15	(Cicco, 2024)	USA	Preventing teacher and counselor burnout: self-care in training programs	Examine the concerns associated with heightened stress levels experienced by education professionals, particularly teachers and counselors-in-training.	435 lecturers	A cross-sectional study	Quantitative research	Tips for self-care that lead to resilience and burnout prevention can potentially be useful for educators.	36
16	(Hoter & Reiner, 2023)	Israel	Preventing academic burnout and ensuring the wellbeing of teachers returning to academic studies	Examine how mature Israeli teachers returning to academia after many years cope with the burden of their masters’ studies in addition to their work as teachers and how the college can improve the well-being of these students and help avoid academic burnout.	18 female teachers	A cross-sectional study	Qualitative research	The results point to the need for more coordination between staff, involving students in academic and administrative decisions and to introduce an ongoing program accompanying the M.Ed. program that includes a support system to help reduce stress and avoid academic burnout	36
17	(Kassim et al., 2020)	Malaysia	How do academicians cope with occupational stressors to alleviate burnout? The experience of a research university	Determine how academicians cope with the various burdens of academia work stressors to overcome burnout.	327 university academicians	A cross-sectional study	Quantitative research	Academicians adopt maladaptive coping for research and interpersonal conflicts to suppress burnout. An integrative approach at both organization and individual levels is crucial to enhance appropriate coping mechanisms to curb burnout among the academicians of a research university	37
18	(Li et al., 2020)	China	The impact of teaching–research conflict on job burnout among university teachers: An integrated model	Explore the mechanism underlying the relationship between Teaching–Research Conflict (TRC) and job burnout among university teachers using the lens of the Job Demands–Resources (JD-R) model.	489 teachers	A cross-sectional study	Quantitative research	TRC was positively linked to Emotional Exhaustion (EE) and Depersonalization (DP), but negatively linked to personal accomplishment; PSS moderated the effect of TRC on both EE and DP but did not act as a moderator in the relationship between TRC and personal accomplishment; and PsyCap moderated the effect of TRC on all the three dimensions of job burnout.	37
19	(Lian et al., 2021)	China	Calling, character strengths, career identity, and job burnout in young Chinese university teachers: A chain-mediating model	Examine whether or not character strengths and calling could influence the job burnout of young Chinese university teachers. Moreover, it also examined the chain-mediating effect of calling and career identity on character strengths and job burnout	447 young university teachers	A cross-sectional study	Quantitative research	Young university teachers should focus on using their character strengths to improve their calling, enhance their career identity, and reduce job burnout.	39
20	(Sözer-Boz et al., 2024)	Turkey	The Relationships between Burnout Profiles, Teacher Agency, and Meaningful Work of Special Education Teachers	Identify burnout profiles of SETs and explore the relationship between these profiles and the levels of teacher agency and meaningful work.	258 lecturers	A cross-sectional study	Quantitative research	The majority of the SETs require support through interventions to reduce burnout symptoms and enhance overall well-being.	36
21	(Taylor & Frechette, 2022)	USA	The impact of workload, productivity, and social support on burnout among marketing faculty during the COVID-19 pandemic	Examine the impact of the pandemic on faculty workloads and subsequent faculty burnout	88 lecturers	A cross-sectional study	Quantitative research	Chairs should facilitate departmental collaborations to both share insights learned from teaching remotely and to provide emotional support and encouragement.	37
